# Factors Associated with HIV/AIDS in Sudan

**DOI:** 10.1155/2013/971203

**Published:** 2013-07-17

**Authors:** Badreldin Abdelrhman Mohamed, Mohamed Salih Mahfouz

**Affiliations:** ^1^Department of Community Health Sciences, College of Applied Medical Sciences, King Saud University, P.O. Box 10219, Riyadh 11433, Saudi Arabia; ^2^Department of Family and Community Medicine, Faculty of Medicine, Jazan University, P.O. Box 2531, Jazan 45142, Saudi Arabia

## Abstract

*Objectives*. To assess participants' knowledge about HIV/AIDS and to identify the factors associated with HIV/AIDS in Sudan. *Methods*. Observational cross-sectional study carried out at Omdurman National Voluntary Counseling and Testing Centre, Sudan covered 870 participants. Sociodemographic data as well as information related to sexual behavior were collected. *Results*. Most of the respondents were knowledgeable about the true transmission modes for AIDS virus. Very few respondents knew someone infected with AIDS (4.5%), died of AIDS (8.1%), accepted to live with someone infected with AIDS (4.7%) or to work with someone infected with AIDS (2.1%). Regarding sexual behavior, 96.5% had reported their first sexual experience between 20 and 30 years, with 85.7% reporting one or two partners, and only 1.8% reported using condom. Multivariate logistic regression showed that circumcision, religion, marital status, age at first sex, number of sexual partners, education level, and misconception of knowledge are the main risk factors associated with HIV/AIDS. *Conclusion*. Our results showed that a number of diversity risk factors were associated with HIV/AIDS. It is unlikely that a holistic approach will be found to immediately change sexual-risk-relating behavior. Interventions including sustained educational programs, promotion of condom, and encouragement of voluntary testing and active involvement of the country's political and religious leaders will be needed to alleviate this problem.

## 1. Introduction

Sudan is bordered by countries with high rates of HIV infection. The first AIDS case was reported in 1986, and by 2011 the total number of cases reported had increased to 2218 [1–3]. In 2002, a behavioral and epidemiological survey (BES) was carried out by Sudan National AIDS Program [1]. According to the survey, the prevalence among general population was 1.6% [1, 4]. Although this survey was the largest survey conducted in Sudan, it was inconclusive largely due to study limitations and mixing of samples that included both general and at-risk populations [4]. 

According to UNAIDS 2011, the most recent estimate of the prevalence of HIV/AIDS in Sudan after South Sudan separation indicated that HIV/AIDS prevalence is around 0.4% [5]. Although this new estimate put Sudan among the lowest countries with HIV/AIDS prevalence in Sub-Saharan Africa, the prevention efforts are far from what is needed. The Sudan House Hold Survey (SHHS) conducted in 2010 estimated that the HIV/AIDS test coverage was only 1% for Sudanese population [4]. 

The spread of HIV is influenced by poverty and illiteracy, both of which are widespread in Sudan. The movement of people displaced by harsh environmental conditions has contributed to an increase in the number of HIV/AIDS cases. Refugees from other conflicts in the region also flee to Sudan. The war in Darfur resulted in the movement of large population fleeing from the war. This resulted in displacement of about 2.5 million people with many contracted the disease [6]. The new war in Blue Nile and South Kordofan also contributed to unfavorable socioeconomic and health situations in Sudan [7].

Sudan is a generally conservative and highly religious society where sexuality is not openly discussed, and a disease such as HIV/AIDS is surrounded by myths and taboos and even ignored in official circles. It is difficult and requires sensitivity to get people to talk openly about the disease. A review of the literature found no previous studies addressing HIV risk factors in Sudan. The main objective of this paper is to assess participants' knowledge about HIV/AIDS and to examine the main factors associated with HIV/AIDS in Sudan.

## 2. Material and Methods

This is observational cross-sectional study conducted using socio-demographic data collected from the records of Omdurman National Voluntary Counseling and Testing (VCT) Centre. According to the WHO (2005) [8], the VCT Centre is ranked as one of the best international centres in the region. The clients of the centre come from different segments of the society and represent all regions of Sudan.

The data used in the analysis cover the period from January 2010 to July 2010. A sample size with the marginal error 2.5% expected prevalence of 1.6% and confidence interval of 95%; nonresponse rate of 10% was calculated for 1016 participants. All the pieces of information have to be collected before the result of the test is obtained. Among the subjects selected, 870 agreed to participate in the study. Cultural inhibitions are behind people's reluctance to indulge in a direct and a free conversation about sex-related questions which are culturally dubbed taboos, especially among females. Thus, information related to sexual behavior was obtained through the “land telephone” or “mobile phone,” as almost all Sudanese have “mobile phones.” After the results were released, we found the sample covered both those with the infection detected (300) and without the infection detected (600) during this period. Many people who come for testing prefer not to return and discover the outcome of the test. 

The data were analyzed using *SPSS* version 17.0. Descriptive statistics (mean, frequencies, and proportion) were used to describe the study variables. The chi-squared test was used to test for associations. The stepwise multiple logistic regression was used to assess the strength of the association of each variable adjusted for other confounders. All the regressor variables were treated as categorical. A *P* value <0.05 was considered statistically significant.

Ethical clearance and permission was obtained from the local authorities of health department at the beginning of the data collection. The study proposal and instrument were approved by King Saud University's review board. Authorization was granted from the Center. During data collection, patients were informed that the information collected would be kept anonymous and that participation was completely voluntary.

## 3. Results

Data were obtained from 870 subjects, giving response rate of 85.6%.  Table 1 shows the sociodemographic characteristics of the selected subjects. The majority of the subjects were males, Muslims, with age less than 39 years, married, with secondary school and university graduate diploma and with income between 500,000 and 900,000 Sudanese pound (SP; 1 dollar = 5600 SP).  Table 2 reports the results of knowledge and attitude about AIDS. All the respondents are aware of the existence of AIDS, with more females reporting that AIDS can be cured and there is vaccine for AIDS. While most of the respondents were knowledgeable about the true transmission mode of AIDS virus, there were many misconceptions about “false” transmission modes. Very few reported that they knew someone infected with HIV (3.9% for males and 5.1% for females) or someone died of AIDS (3.7% for males and 5.8% for females). Very few accepted to live in the same household or work in the same place with someone affected with AIDS. Regarding perception of risk, females perceived themselves not to be at risk at all or have small risk.  Table 3 displays sexual behavior among sexually active respondents. All of the respondents reported having sexual affair in the preceding six months. Most males and females reported their first sexual experience to be between the ages of 20 and 25. The mean age of the first sexual experience for males was 21.6 years and for females was 24.8 years. The answer to the question “number of sexual partners in the last 6 months” showed that the majority of males reported they had two sexual partners (78.3%) with mostly type of partner as lover (51.1%) and only 3.5% used condom in the last sexual intercourse. For females, the majority reported one sexual partner (49.3%) with mostly type of partner as lover (92.8%). Most of the sexual partners were not relatively close in age; 77% of males and 79.5% of females reported an age difference of 5–14 years. Stepwise multiple regression (Table 4) showed that circumcision, religion, marital status, age at first sex, number of sexual partners, education, and misconception knowledge are important risk factors associated with HIV.

## 4. Discussion

In a very conservative Muslim community like Sudan, people with AIDS are seen as ignominious. Stigma towards people with HIV is prevalent due to ignorance, lack of awareness, and misconception about transmission of the disease (Table 2), and most people think that the disease is connected with homosexuals, sex workers, and drug users. The lack of awareness with regard to HIV/AIDS is mainly due to the insufficient government commitment towards public health education, the weak role of the media, and also the government's educational policies, which favor unplanned expansion in admissions over quality, and content of curriculums particularly at the secondary and the higher educational levels. In such a community, families are shunned, discriminated isolated if they are associated with someone with AIDS and families hide such information. It is not surprising that 95.5% of the respondents did not know someone with AIDS, 91.95% not knowing someone died with AIDS, and 84% not accepting living with someone with AIDS in the same household. Those who know their diagnosis often devise ways of hiding this out of fear and shame, and some may even seek revenge by spreading out the HIV [9].

Age at first sexual intercourse, when it is before marriage, is associated with longer exposure to sexual activity and higher likelihood of accumulating sexual partners [10]. Individuals are most likely to have multiple partners during the period between the first intercourse and first marriage [11]. The median age at marriage is 29.1 years for males and the median age at first sexual intercourse is 22.4 years. This long period exposure to sexual activity is more likely to be correlated with higher risk infections with HIV/AIDS. For female median age at marriage, also increased during the past years in Sudan, due to the expansion in women education in Sudan [12]. The exposure to “Western culture” influence which has been aided by mass media entertainment products, such as sex films, video clubs, private channels, internet, and advertising, is perceived as portraying sexual behavior as a normal and recreational activity without restrictions. 

The majority (98%) in our sample resist using the condom. This resistance is due to the lack of sufficient public health education; the use of condom has been viewed as a method of contraception more than a method to prevent sexually transmitted diseases and HIV/AIDS. Added to that is the problem of getting condoms. Condoms are only available in some pharmacies and are very costly, and even in those pharmacies are kept under the counter and only dispensed to those who ask for them.

Our results about AIDS knowledge revealed that while all respondents had heard of AIDS, more than 60% did not believe that a healthy-looking person can carry the AIDS virus. This belief can lead to exposure to HIV infection since people can unlikely take precautions when having sexual intercourse with healthy-looking partners. We found more than 75% believe that mosquitoes transmit the HIV virus; those people may see the use of condom as futile. The belief that AIDS is a disease for “high-risk” group can influence people's perception and behavior. As a result, some people take their own risk because they did not identify these high-risk groups. Some see AIDS as punishment of immoral behavior so that those who see their lifestyle as being morally upright may see their chance of being infected by HIV is very low. Myth about cure of AIDS has been found in different Sudanese populations. About 48% believe that AIDS can be cured. Such misconception may encourage some persons to practice risky behavior by creating a false impression on them that they will be cured if they become infected with HIV. 

HIV transmission risk is not simple to be characterized or contained. There are multitudes of demographic, social, and biological factors that contribute to such risk. The findings of this study showed that circumcision, religion, marital status, age at first sex, education level, number of sexual partners, and misconception of HIV knowledge are significant predictors for HIV infections. These findings should be considered in any intervention strategy in the country.

The most prevalent type of circumcision among females involves tight suturing of the cut edges of the labia majora, leaving only a very small opening, just large enough for passage of urine and menstrual blood. The prevalence of circumcision declined from 89.2% in 2000 to 69.4% in 2006; it is surprising that 65% reported being circumcised in our study [13]. Vaginal intercourse is difficult, often resulting in tissue damage, lesions, and postcoital bleeding. These tears would tend to make squamous vaginal epithelium similar in permeability to the columnar muscosa of the rectum, thus facilitating the possible transmission of HIV [14]. A great deal of anal sexual intercourse takes place in cases where the female is circumcised [15]. Male circumcision reduces the risk of HIV infection but only provides partial protection [16]. It is not a substitute for other proven HIV prevention methods. In our study, we found about 2% were using condom, and most of the respondents were sexually active.

Various studies in Africa have shown that male partners' sexual behavior is a major determinant of HIV infection among women not engaging in high-risk sexual practices [17] Prostitution is illegal and, therefore, clandestine. This makes it difficult to determine the true extent of the sex work industry, although it is acknowledged substantial and has apparently been increasing in recent years mainly due to socioeconomic factors. Many married men resort to infidelity because of the practice of the postpartum sexual abstinence [18]. In Sudan, the period of postpartum abstinence is long, ranging from six to 10 weeks. 

Behavioral data for our study was conducted using self-administered questionnaire, so denial of use is expected from some participants. Therefore, the results of our study should be interpreted with caution. Also, the study was based on a cross-sectional study design, which is not enough for generating strong associations between risk factors and HIV/AIDS.

## 5. Conclusion and Recommendation

Our results provided new information regarding potential risk factors for contracting HIV. However, since this is the first study in Sudan, we included too many factors. We hope that future research addresses those factors separately and in depth. The prevention and control of HIV infections to be successful needs clear strategies to prevent new cases of the disease and offer proper treatment to all those currently infected persons. Voluntary HIV testing programs can be very useful in this regard as they can effectively reduce risk behaviors among those at higher risk. 

## Figures and Tables

**Table 1:** Sociodemographic characteristics of study participants.

Characteristic	*N* (%)
Religion	
Muslims	712 (81.8)
Non Muslims	158 (18.2)
Age	
20–29	320 (36.8)
30–39	278 (32.0)
40–49	142 (16.3)
≤50	130 (14.9)
Gender	
Male	456 (52.4)
Female	414 (47.6)
Marital status	
Single	339 (39.0)
Married	434 (49.9)
Divorced	97 (11.1)
Education level	
Illiterate	106 (12.2)
Primary	187 (21.5)
Secondary	291 (33.5)
University and above	286 (32.8)
Income (SP)	
100,000–<300,000	121 (15.1)
300,000–<500,000	104 (12.0)
500,000–<700,000	228 (26.1)
700,000–<900,000	253 (30.0)
≥9000,000	164 (16.8)
Circumcisions	
Females	
Yes	270 (65.2)
No	144 (34.8)
Males	
Yes	100 (456)

**Table 2:** AIDS knowledge and attitude among respondents.

Item	Male *N* (%)	Female *N* (%)	Total *N* (%)
Knowledge			
Heard of HIV	456 (100)	414 (100)	870 (100)
AIDS can be cured	112 (24.6)	308 (74.4)	420 (48.3)
There is a vaccine for AIDS	110 (24.1)	212 (51.2)	323 (37.1)
True transmission route			
Sexual contact	448 (98.2)	411 (99.3)	859 (98.7)
Blood transfusion	442 (96.9)	338 (81.6)	780 (89.6)
Mother to child	256 (56.1)	376 (90.8)	632 (72.7)
Shaver blade of the infected	401 (87.9)	252 (60.9)	653 (75.7)
Sharing of needles	413 (90.6)	176 (42.5)	589 (67.7)
False transmission route			
Sharing food with the infected	319 (70.0)	191 (46.1)	510 (58.6)
Using public toilets	428 (93.9)	174 (42.0)	602 (69.2)
Being coughed or sneezed on	201 (44.1)	218 (52.7)	419 (48.2)
Mosquitoes bites	325 (71.3)	337 (81.4)	662 (76.1)
Shaking hands with the infected	237 (52.0)	409 (98.8)	646 (74.3)
Healthy-looking person cannot have HIV	231 (50.7)	203 (49.0)	534 (61.4)
Swimming places	388 (85.1)	293 (70.8)	631 (72.5)
Know anyone affected by AIDS			
Yes	18 (03.9)	21 (05.1)	039 (04.5)
No	438 (96.1)	393 (91.9)	831 (95.5)
Know anyone died of AIDS			
Yes	39 (08.6)	31 (07.5)	70 (08.1)
No	417 (91.4)	383 (92.5)	800 (91.9)
Do you accept living in the same household with a person infected with AIDS?			
Yes	17 (03.7)	24 (05.8)	041 (04.7)
No	381 (83.6)	354 (85.5)	733 (84.3)
I do not know	58 (20.6)	36 (08.7)	94 (11.0)
Do you accept working in the same place with a person infected with AIDS?			
Yes	18 (03.9)	1 (00.3)	19 (02.1)
No	358 (78.5)	311 (75.1)	668 (76.7)
I do not know	81 (17.8)	102 (24.6)	183 (21.2)
Perceived risk of HIV			
(1) None	96 (21.1)	144 (34.8)	240 (27.6)
(2) Small	172 (37.7)	163 (39.4)	335 (38.5)
(3) Moderate	174 (38.1)	99 (23.9)	273 (31.4)
(4) Great	14 (03.1)	8 (01.9)	22 (02.5)

**Table 3:** Sexual behavior among sexually active respondents.

Characteristics	Male *N* (%)	Female *N* (%)	*N* (%)	*P* value
Age at first sex				
<20	17 (03.7)	0	17 (01.9)	0.001
20–<25	301 (66.1)	174 (42.0)	475 (54.6)	
25–≤30	137 (30.0)	228 (55.1)	365 (41.9)	
>30	1 (0.2)	12 (02.9)	13 (01.6)	
Mean age at first sex (year)	21.6	24.8	22.4	
Number of sexual partners in the last 6 months				
1	34 (07.5)	204 (49.3)	238 (27.3)	0.0001
2	357 (78.3)	151 (36.5)	508 (58.4)	
3	51 (11.2)	59 (14.2)	110 (12.6)	
≥4	14 (0.31)	0 (0)	14 (01.7)	
Type of partner				
(1) Lover	233 (51.1)	384 (92.8)	617 (70.9)	0.0001
(2) Casual	223 (48.9)	30 (7.2)	253 (29.1)	
Age difference for recent partner (years)				
<5	83 (18.2)	21 (05.1)	104 (12.0)	0.0001
5–14	351 (77.0)	329 (79.5)	680 (68.0)	
15–≤25	15 (3.3)	43 (10.3)	58 (16.9)	
More than 25	7 (1.5)	21 (05.1)	28 (03.1)	
Use condom at last sexual intercourse				
Yes	16 (3.5)	0 (0.0)	16 (01.8)	—
No	440 (96.5)	414 (100.0)	854 (98.2)	

**Table 4:** Stepwise multiple logistic regression of predictors for risk variables.

Variable	Odds ratio	*P* value	95% CI
Marital status			
Single	1		
Married	2.32	0.027	1.09–3.42
Divorced	1.68	0.011	1.02–3.21
Religion			
Non-Muslims	1		
Muslims	0.36	0.003	0.15–0.93
Circumcision			
No	1		
Yes	2.41	0.014	1.13–3.28
Age at first sex			
<20	1		
20–<25	1.58	0.014	1.05–3.58
25–≤30	0.86	0.082	0.37–1.59
>30	0.47	0.037	0.01–0.93
Number of sexual partners			
1	1		
2	1.03	0.087	0.48–2.33
3	3.51	0.004	1.28–5.68
≥4	4.22	0.001	2.15–5.72
Misconception knowledge			
Yes	1		
No	2.34	0.012	1.39–4.34
Education level			
Illiterate	1		
Elementary and intermediate	1.02	0.43	0.28–3.41
Secondary	0.97	0.27	0.35–2.89
University and above	0.46	0.006	0.02–1.31
